# Supplementary material for: Identification of Novel microRNA Prognostic Markers Using Cascaded Wx, a Neural Network-Based Framework, in Lung Adenocarcinoma Patients
Source: Cancers (Basel). 2020 Jul 14;12(7):1890. doi: 10.3390/cancers12071890 (PMC7409139; doi:10.3390/cancers12071890)
Supplement: Supplementary file 1 [file cancers-12-01890-s001.pdf]

cancers

Article

Identification of Novel microRNA Prognostic Markers Using Cascaded Wx, a Neural Network-Based Framework, in Lung Adenocarcinoma Patients

Jeong Seon Kim, Sang Hoon Chun, Sungsoo Park, Sieun Lee, Sae Eun Kim, Ji Hyung Hong, Keunsoo Kang, Yoon Ho Ko and Young-Ho Ahn

Supplementary Materials:

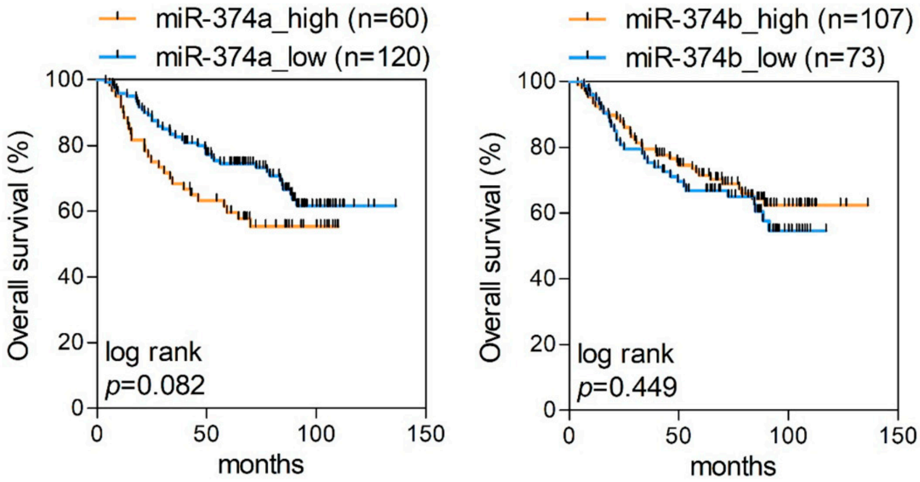

Figure S1. miR-374 and miR-374b are poor prognostic markers in LUAD patients. Overall survival of LUAD patients based on miR-374a and miR-374b expression. LUAD patients were divided into two groups (high and low) based on expression levels of miR-374a (left) or miR-374b (right) obtained from NanoString.

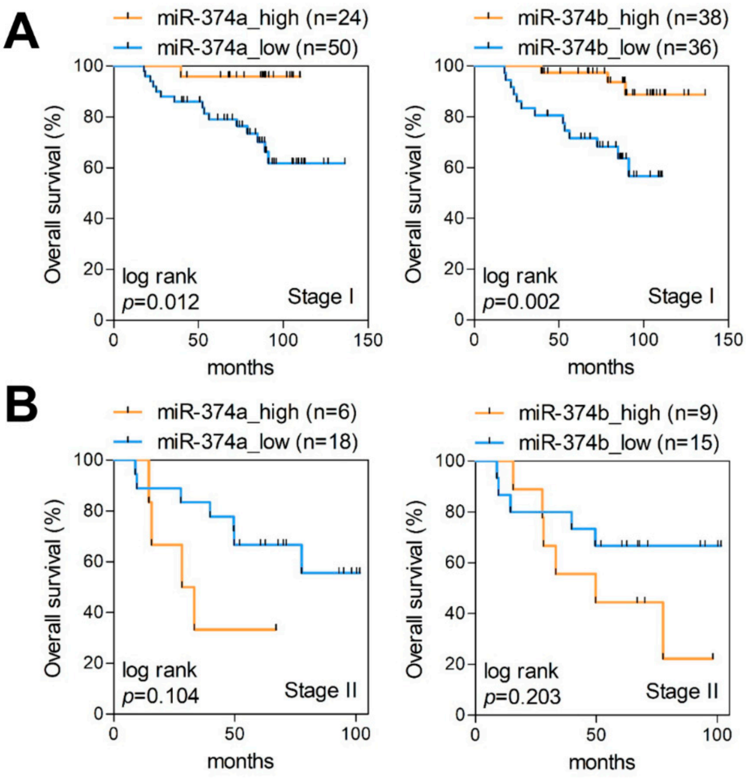

Figure S2. The effects of miR-374a and miR-374b on patient overall survival are cancer stage-specific. (**A**) Overall survival of stage I-LUAD patients based on miR-374a and miR-374b expression. Patients (n = 74) were divided into two groups (high and low) based on the expression levels of miR-374a (left) or miR-374b (right) obtained from NanoString. (B) Overall survival of stage II-LUAD patients based on miR-374a and miR-374b expression. Patients (n = 24) were divided into two groups (high and low) based on the expression levels of miR-374a (left) or miR-374b (right) obtained from NanoString.

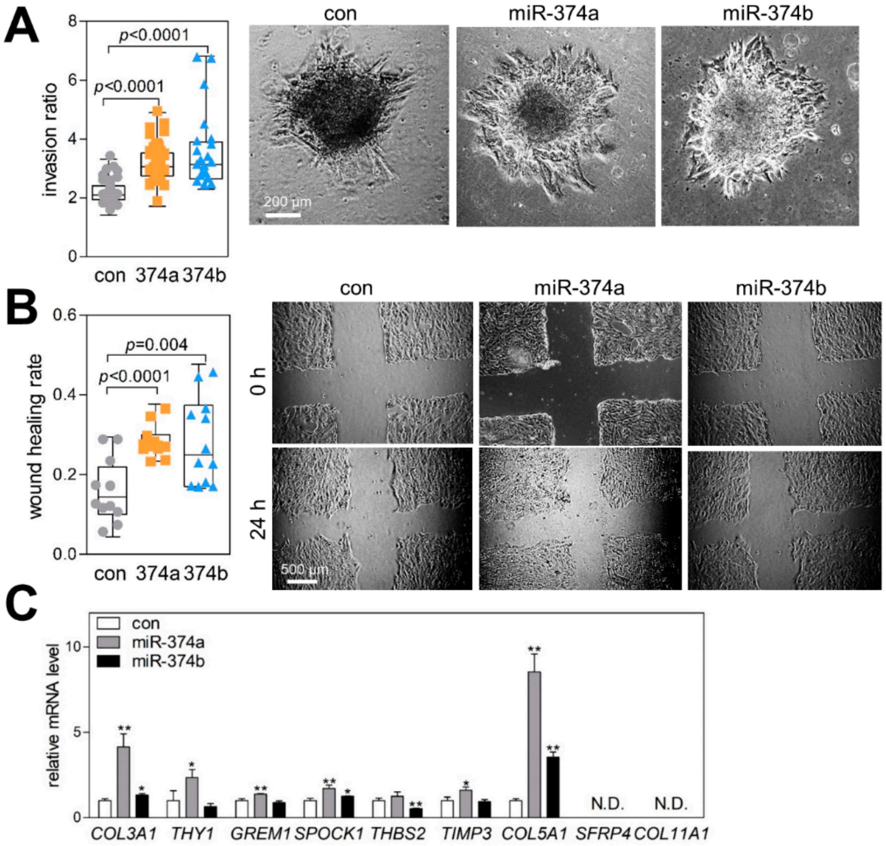

Figure S3. miR-374a and miR-374b promote invasion of H1792 lung cancer cells. (**A**) Spheroid invasion assay in miR-374 mimic-transfected cells. Spheroids made from hanging-drop cultures of miR-374 mimic-transfected H1792 cells were seeded on collagen gels and then cultured for 24 h. Spheroid invasion ratios (ratio of whole cell area to central spheroid area) were measured using ImageJ. Box-and-whisker plots denote median and upper/lower quartiles+1.5×IQR (interquartile range). con, n = 45; 374a, n = 42; 374b, n = 20. *p*, two-tailed Student's t-test. (**B**) Wound-healing assay in miR-374 mimic-transfected H1792 cells. Cells were subjected to scratch wounds and then incubated for 24 h with mitomycin C (1 μg/mL) to block proliferation-related effect. Wound healing rates (1−[wound area ratio of 24 h to 0 h]) were measured using ImageJ (n = 12). *p*, two-tailed Student's *t*-test. (**C**) qRT-PCR of EMT and invasiveness signature genes in miR-374 mimic-transfected cells. H1792 cells were transiently transfected with miR-374a or miR-374b mimic, and expression levels of the signature genes were measured by qRT-PCR. Expression levels were normalized to *RPL32* level. Relative values to those of H1792 cells transfected with negative control (set at 1.0) are presented. Data are mean+SD (n = 3). *$p < 0.05$, ** $p < 0.01$; two-tailed Student's t-test. N.D., not detected.

cancers MDPI

Table S1. The CWx miRNA list.

rank	miRNAs	CWx score	rank	miRNAs	CWx score	rank	miRNAs	CWx score
1	hsa-mir-374a	466	67	hsa-mir-183	155	133	hsa-mir-1301	49
2	hsa-mir-374b	448	68	hsa-mir-505	152	134	hsa-mir-452	49
3	hsa-let-7f-1	436	69	hsa-mir-155	150	135	hsa-mir-3130-1	48
4	hsa-mir-101-1	421	70	hsa-mir-34a	149	136	hsa-mir-19b-1	46
5	hsa-mir-21	415	71	hsa-mir-421	148	137	hsa-mir-146b	46
6	hsa-mir-22	398	72	hsa-mir-3607	147	138	hsa-mir-1254	43
7	hsa-mir-15a	386	73	hsa-mir-106a	146	139	hsa-mir-132	43
8	hsa-mir-141	386	74	hsa-mir-125b-2	145	140	hsa-mir-134	43
9	hsa-mir-98	376	75	hsa-mir-582	145	141	hsa-mir-199a-1	42
10	hsa-mir-182	367	76	hsa-mir-375	141	142	hsa-mir-550a-2	41
11	hsa-mir-142	364	77	hsa-mir-628	140	143	hsa-mir-625	39
12	hsa-mir-146a	349	78	hsa-mir-152	136	144	hsa-mir-196b	38
13	hsa-mir-218-2	346	79	hsa-mir-3677	135	145	hsa-mir-140	37
14	hsa-mir-200c	343	80	hsa-mir-429	134	146	hsa-mir-20b	37
15	hsa-let-7f-2	340	81	hsa-mir-9-2	131	147	hsa-mir-2277	35
16	hsa-mir-708	332	82	hsa-mir-19a	126	148	hsa-mir-378c	35
17	hsa-mir-29b-1	331	83	hsa-mir-503	126	149	hsa-mir-3117	34
18	hsa-mir-32	327	84	hsa-mir-511-2	125	150	hsa-mir-181d	34
19	hsa-mir-590	310	85	hsa-mir-301a	119	151	hsa-mir-33a	34
20	hsa-mir-19b-2	309	86	hsa-mir-92b	118	152	hsa-mir-1266	33
21	hsa-mir-210	303	87	hsa-mir-27b	116	153	hsa-mir-548b	32
22	hsa-mir-20a	300	88	hsa-mir-143	113	154	hsa-mir-9-1	31
23	hsa-mir-128-1	276	89	hsa-mir-1307	112	155	hsa-mir-581	29
24	hsa-mir-17	257	90	hsa-mir-379	110	156	hsa-mir-424	29
25	hsa-let-7g	257	91	hsa-mir-203	109	157	hsa-mir-145	28
26	hsa-mir-101-2	256	92	hsa-mir-148b	107	158	hsa-mir-3614	27
27	hsa-mir-335	252	93	hsa-mir-103-2	103	159	hsa-let-7b	27
28	hsa-mir-148a	250	94	hsa-mir-338	103	160	hsa-mir-942	27
29	hsa-mir-200b	250	95	hsa-mir-339	103	161	hsa-mir-223	26
30	hsa-mir-542	249	96	hsa-mir-769	101	162	hsa-mir-337	25
31	hsa-mir-100	249	97	hsa-mir-511-1	98	163	hsa-mir-382	24
32	hsa-mir-454	248	98	hsa-mir-191	98	164	hsa-mir-501	24
33	hsa-mir-128-2	242	99	hsa-mir-24-2	97	165	hsa-mir-1255a	24
34	hsa-mir-4326	236	100	hsa-mir-195	94	166	hsa-mir-361	23
35	hsa-mir-2355	234	101	hsa-mir-212	91	167	hsa-mir-362	22
36	hsa-mir-29a	234	102	hsa-mir-99a	90	168	hsa-mir-450a-1	22
37	hsa-mir-340	228	103	hsa-mir-330	90	169	hsa-mir-221	21
38	hsa-mir-107	224	104	hsa-mir-30a	89	170	hsa-mir-30c-1	20
39	hsa-mir-3913-1	223	105	hsa-mir-127	88	171	hsa-mir-181a-1	18
40	hsa-let-7a-2	218	106	hsa-mir-3136	88	172	hsa-mir-1293	18
41	hsa-let-7a-3	217	107	hsa-mir-1-2	86	173	hsa-mir-3065	17

42	hsa-mir-103-1	214	108	hsa-mir-126	85	174	hsa-let-7i	17
43	hsa-let-7a-1	208	109	hsa-let-7c	84	175	hsa-mir-760	17
44	hsa-mir-135b	207	110	hsa-mir-598	82	176	hsa-mir-381	16
45	hsa-mir-29c	207	111	hsa-mir-29b-2	82	177	hsa-mir-939	16
46	hsa-mir-23b	206	112	hsa-mir-3613	79	178	hsa-mir-3682	16
47	hsa-mir-151	197	113	hsa-mir-451	77	179	hsa-mir-3934	14
48	hsa-mir-7-1	191	114	hsa-mir-550a-1	75	180	hsa-mir-1287	12
49	hsa-mir-26a-2	189	115	hsa-mir-589	73	181	hsa-mir-194-1	11
50	hsa-let-7e	188	116	hsa-mir-144	72	182	hsa-mir-654	11
51	hsa-mir-497	184	117	hsa-mir-199a-2	71	183	hsa-mir-450a-2	10
52	hsa-mir-96	177	118	hsa-mir-425	70	184	hsa-mir-181b-2	9
53	hsa-mir-181a-2	176	119	hsa-mir-219-1	70	185	hsa-mir-324	9
54	hsa-mir-629	174	120	hsa-mir-1274b	67	186	hsa-mir-214	9
55	hsa-mir-130b	174	121	hsa-mir-23a	67	187	hsa-mir-33b	8
56	hsa-mir-150	174	122	hsa-mir-455	65	188	hsa-mir-491	6
57	hsa-mir-200a	174	123	hsa-mir-181c	65	189	hsa-mir-130a	6
58	hsa-mir-27a	165	124	hsa-mir-624	64	190	hsa-mir-192	6
59	hsa-mir-31	165	125	hsa-mir-660	64	191	hsa-mir-500b	5
60	hsa-mir-16-1	163	126	hsa-mir-10a	64	192	hsa-mir-153-2	4
61	hsa-mir-181b-1	162	127	hsa-mir-3158-2	63	193	hsa-mir-10b	4
62	hsa-mir-93	161	128	hsa-mir-30d	62	194	hsa-mir-185	4
63	hsa-mir-18a	159	129	hsa-mir-125b-1	60	195	hsa-mir-320b-2	3
64	hsa-mir-450b	159	130	hsa-mir-34b	54	196	hsa-mir-186	2
65	hsa-mir-199b	158	131	hsa-mir-342	53	197	hsa-mir-576	1
66	hsa-mir-30e	156	132	hsa-mir-26b	51			

cancers MDPI

Table S2. Univariate and multivariate analysis using the Cox proportional hazards model for recurrence-free survival.

Variables	Univariate			Multivariate		
	HR	95% CI	*p*-value	HR	95% CI	*p*-value
Gender (**female** vs. male)	1.165	0.722–1.881	0.532			
Age (**>65** vs. ≤65)	1.215	0.752 – 1.963	0.426			
Differentiation(**poor/mod** vs. good)	2.376	1.243–4.539	0.009	1.583	0.794–3.155	0.192
T stage (**T3/4** vs. T1/2)	2.380	1.297–4.368	0.005	1.481	0.791–2.773	0.220
N stage (**N1-3** vs. N0)	3.974	2.444–6.461	< 0.001	1.757	0.925–3.340	0.085
Lymphatic invasion (**yes** vs. no)	1.139	1.007–1.288	0.039	2.424	1.276–4.605	0.007
Vascular invasion (**yes** vs. no)	1.067	0.913–1.248	0.412			
Peri-neural invasion (**yes** vs. no)	1.042	0.876–1.239	0.642			
High miR-374a (**high** vs. low)	1.593	0.977–2.596	0.062	1.642	0.995–2.709	0.053
High miR-374b (**high** vs. low)	1.568	0.936–2.628	0.088	1.218	0.713–2.080	0.471

Table S3. Univariate and multivariate analysis using the Cox proportional hazards model for overall survival.

Variables	Univariate			Multivariate		
	HR	95% CI	*p*-value	HR	95% CI	*p*-value
Gender (female vs. male)	0.828	0.508–1.351	0.450			
Age (>65 vs. ≤65)	1.332	0.818–2.170	0.249			
Differentiation(poor/mod vs. good)	1.114	0.787 – 1.576	0.175			
T stage (T3/4 vs. T1/2)	1.957	1.020–3.758	0.044	1.253	0.631–2.487	0.520
N stage (N1-3 vs. N0)	2.774	1.676–4.590	< 0.001	1.482	0.750–2.927	0.258
Lymphatic invasion (yes vs. no)	1.235	1.108–1.378	< 0.001	2.317	1.388–3.868	0.001
Vascular invasion (yes vs. no)	1.199	1.066–1.349	0.002	1.072	0.465–2.470	0.871
Peri-neural invasion (yes vs. no)	1.189	1.053–1.341	0.005	1.886	0.571–6.229	0.298
High miR-374a (high vs. low)	1.548	0.942–2.544	0.085	1.804	1.084–3.003	0.023
High miR-374b (high vs. low)	0.828	0.508–1.350	0.449			

Table S4. Sequences of qRT-PCR primers used in this study.

Genes	Forward (5′→3′)	Reverse(5′→3′)
RPL32	ACAAAGCACATGCTGCCCAGTG	TTCCACGATGGCTTTGCGGTTC
SNAI1	CTGAGGCCAAGGATCTCCAG	ATCTGCGGCAAGGCGTTTTCCA
SNIA2	ATCTGCGGCAAGGCGTTTTCCA	GAGCCCTCAGATTTGACCTGTC
CDH1	GCCTCCTGAAAAGAGAGTGGAAG	TGGCAGTGTCTCTCCAAATCCG
INADL	ACAAGGCAGATTTGACGACCTGG	CTTTGAGCCACAACAGGAAGGTC
CRB3	CTTCTGCAAATGAGAATAGCACTG	GACCACGATGATAGCAGTGATGG
SPOCK1	GTTCTACTGGCAAAAGCCTCGC	AGGTTCCGCAACTCCTTGTCTG
COL3A1	TGGTCTGCAAGGAATGCCTGGA	TCTTTCCCTGGGACACCATCAG
TIMP3	TACCGAGGCTTCACCAAGATGC	CATCTTGCCATCATAGACGCGAC
COL5A1	GGAGATGATGGTCCCAAAGGCA	CCATCATCTCCTTTGTCACCAGG
SFRP4	CTATGACCGTGGCGTGTGCATT	GCTTAGGCGTTTACAGTCAACATC
THBS2	CAGTCTGAGCAAGTGTGACACC	TTGCAGAGACGGATGCGTGTGA
COL11A1	ATGGACCAGCAGGATTACGTGG	TGTACCTGCTGACCCACGTTCT
GREM1	TCATCAACCGCTTCTGTTACGGC	CAGAAGGAGCAGGACTGAAAGG

© 2020 by the authors. Submitted for possible open access publication under the terms and conditions of the Creative Commons Attribution (CC BY) license (http://creativecommons.org/licenses/by/4.0/).
